# Risk-factors for methadone-specific deaths in Scotland’s methadone-prescription clients between 2009 and 2013*

**DOI:** 10.1016/j.drugalcdep.2016.08.627

**Published:** 2016-10-01

**Authors:** Lu Gao, Polyxeni Dimitropoulou, J. Roy Robertson, Stuart McTaggart, Marion Bennie, Sheila M. Bird

**Affiliations:** a MRC Biostatistics Unit, Cambridge CB2 0SR, United Kingdom; b Usher Institute of Population Health Sciences and Informatics, Edinburgh University, EDINBURGH EH16 4UX, United Kingdom; c Information Services Division, NHS National Services Scotland, Edinburgh EH12 9EB, United Kingdom; d Strathclyde Institute of Pharmacy and Biomedical Sciences, University of Strathclyde, Glasgow G4 0RE, United Kingdom; e Department of Mathematics and Statistics, Strathclyde University, Glasgow G1 1XH, United Kingdom

**Keywords:** Deaths, Drugs-related, Methadone-specific, Risk-factors, Gender, Age-group, Prescribed-methadone, Quantity, Daily-dose, Quintiles

## Abstract

**Aim:**

To quantify gender, age-group and quantity of methadone prescribed as risk factors for drugs-related deaths (DRDs), and for methadone-specific DRDs, in Scotland’s methadone-prescription clients.

**Design:**

Linkage to death-records for Scotland’s methadone-clients with one or more Community Health Index (CHI)-identified methadone prescriptions during July 2009 to June 2013.

**Setting:**

Scotland’s Prescribing Information System and National Records of Scotland.

**Measurements:**

Covariates defined at first CHI-identified methadone prescription, and person-years at-risk (pys) thereafter until the earlier of death-date or 31 December 2013. Methadone-specific DRDs were defined as: methadone implicated but neither heroin nor buprenorphine. Hazard ratios (HRs) were assessed using proportional hazards regression.

**Findings:**

Scotland’s CHI-identified methadone-prescription cohort comprised 33,128 clients, 121,254 pys, 1,171 non-DRDs and 760 DRDs (6.3 per 1,000 pys), of which 362 were methadone-specific. Irrespective of gender, methadone-specific DRD-rate, per 1,000 pys, was higher in the 35+ age-group (4.2; 95% CI: 3.6–4.7) than for younger clients (1.9; 95% CI: 1.5–2.2). For methadone-specific DRDs, age-related HRs (e.g., 2.9 at 45+ years; 95% CI: 2.1–3.9) were steeper than for all DRDs (1.9; 95% CI: 1.5–2.4); there was no hazard-reduction for females; no gender by age-group interaction; and, unlike for all DRDs, the highest quintile for quantity of prescribed methadone at cohort-entry (>1960 mg) was associated with increased HR (1.8; 95% CI: 1.3–2.5).

**Conclusion:**

Higher methadone-specific DRD rates in older clients, irrespective of gender, call for better understanding of methadone’s pharmaco-dynamics in older, opioid-dependent clients, many with progressive physical or mental ill-health.

## Highlights

• Powerful analysis of a national cohort’s 362 methadone-specific deaths.
• Scottish cohort of 33,128 methadone-prescription clients in 2009–2013.
• Steeper age-gradient than for all drugs-related deaths.
• No gender differential in the hazard of methadone-specific deaths.
• Increased hazard at the highest quintile for quantity of methadone prescribed.

## 1 Introduction

Despite other options (buprenorphine, morphine, heroin) methadone, prescribed as a 1 mg/1 ml solution, is the major contributor to opioid substitution therapy (OST) globally and accounts for the vast majority of Scotland’s OST over the past 30 years (World Health Organization, 2009, Department of Health et al., 1999, Department of Health et al., 2007, National Institute for Health and Clinical Excellence, 2007, Information Services Division and Scotland, 2015). With a population of 5.2 million, including 60,000 problem drug users, Scotland dispensed around 600,000 L of methadone per annum from 2009 to 2013. The Scottish Drug Strategy Delivery Commission (2013), tasked by Scottish Ministers to conduct an independent review of Scotland’s OST, endorsed the professional standards (including supervision) by which opioid dependency was managed in Scotland and also the value of methadone as a treatment which halves clients’ risks of drugs-related death (DRD) and reduces blood-borne viruses and criminality (Strang et al., 2010, Degenhardt et al., 2011, White et al., 2015, Pierce et al., 2016, Pierce et al., 2015b).

Over the past decade, different study methods in different jurisdictions have persuaded the addiction community that there is a strong empirical interaction between gender and age-group in DRD-rates, whereby female opioid users’ experience a much lower DRD-rate than their male counterparts at younger ages but, progressively, this advantage weakens beyond 35 years of age. We conjectured that the prescribing of methadone could be implicated in this gender by age-group interaction.

Methadone prescribing was under scrutiny in Scotland because DRDs in which methadone was implicated were increasing, even allowing for the quantity prescribed (as proposed by Strang et al., 2010) and because only a minimum estimate for Scotland’s number of methadone-clients could be given (The Scottish Government, 2008, HM Government, 2010, Ferguson, 2012, Scottish Public Health Observatory, 2015): latterly, those identifiable by their Community Health Index (CHI) number (Ellison, 2015). A CHI-number is uniquely assigned to patients in Scotland’s National Health Service (NHS) and used as a basis for efficient, trusted record-linkage (Pavis and Morris, 2015). However, the availability of the CHI-number on methadone prescriptions varies by prescription-source (Dimitropoulou et al., 2016, Alvarez-Madrazo et al., 2016), being higher for prescriptions issued by general practitioners (GPs) than for other-source prescriptions which are used by community drug services. Using CHI-numbers and plausible assumptions, Dimitropoulou et al. (2016) developed an upper bound for Scotland’s number of methadone-clients: from July 2012 to June 2013 (hereafter 2012/13), 16,900 to 19,600 were managed by Scotland’s GPs but the totality of methadone-clients was 25,100 to 36,500. Greater precision was possible for GP-clients because, by 2012/13, the majority (86%) of methadone prescriptions by GPs had the client’s CHI-number in contrast to 47% for other-source prescriptions.

Questions were raised, and continue to be in the media (Ferguson, 2012, Ellison, 2015), about the persistence of Scotland’s methadone clients across prescription-years, which was substantial during 2009/10 to 2012/13 (see Supplementary material.^1^).

People on longer-term methadone maintenance are, of course, ageing and their DRD-rate rises accordingly (Pierce et al., 2016, Merrall et al., 2012, Pierce et al., 2015a, King et al., 2013, King et al., 2014, Cousins et al., 2011, Cornish et al., 2010, Bird et al., 2010).

We hypothesised that possible reasons for OST clients’ increasing DRD-rate with age included the methadone prescribed to older clients (Dimitropoulou et al., 2016), as directly implicated or indirectly through co-morbidities. Electrocardiograms are recommended for older or persistent methadone clients on higher doses (Medicines and Healthcare products Regulatory Agency (MHRA), 2006) to detect QTc prolongation (see Discussion section, below), for which being female (Makkar et al., 1993, Kest et al., 2000, Lee and Ho, 2013, Graziani and Nistico, 2015, Bawor et al., 2014), co-morbidities and co-prescribing are also risk factors.

As access to OST has expanded, the opioid-specificity of DRDs in Scotland, as elsewhere, has changed (Strang et al., 2010, Zador and Sunjic, 2002, Seymour et al., 2003, McCowan et al., 2009, Bird and Robertson, 2011, Pilgrim et al., 2013, Bernard et al., 2013, Cousins et al., 2016, Winker et al., 2014, Kimber et al., 2015). National Records of Scotland (NRS, 2015) now publishes, as official statistics, Scotland’s number of opioid-related deaths cross-tabulated by gender and age-group. Co-presence of benzodiazepines or alcohol at DRDs has been remarked upon (Seymour et al., 2003, McCowan et al., 2009, Bird and Robertson, 2011, Christie, 2011, Leece et al., 2015, Park et al., 2015) but, whereas diazepam was present at 70% of Scotland’s DRDs in 2013 or 2014, diazepam was implicated in only 14% of Scotland’s DRDs; and, when present, was prescribed in the past 30 days for only one in six DRDs (Barnsdale et al., 2015, Barnsdale et al., 2016) and so was mainly illicitly acquired.

The recent rise in Scotland’s methadone-implicated DRDs, and likewise in England (White and Hamilton, 2016), re-ignited concern about long-term treatment with methadone or its possible diversion. Scotland’s National Drugs-Related Death Database (Barnsdale et al., 2015) reported that methadone was not prescribed at the time of death in 2013 for 41% of those in whose DRD methadone was implicated. Too low a daily-dose of methadone, of course, compromises retention in OST (Kimber et al., 2010) and, since being off-OST doubles clients’ DRD-risk, the public health challenge in 2016, as in 1999 (Ward et al., 1999), is to deliver safe and effective forms of OST for as long and to as many ageing opioid-dependent individuals as can benefit (Department of Health et al., 2007).

Hence, in this paper, our aims were:

1 To document DRD-rates and methadone-specific DRD rates by gender and age-group for Scotland’s cohort of CHI-identified methadone clients between July 2009 and June 2013;
2 To apply proportional hazards regression to analyse risk factors at cohort-accrual (including quintile for the quantity of prescribed methadone) for methadone-specific DRDs; and for all DRDs.

## 2 Methods

### 2.1 Drug related deaths

We used the UK harmonized definition of DRD (National Records of Scotland, 2015) and requested information on the opioid-specificity of Scotland’s DRDs from NRS as follows:

i) Methadone-specific DRDs: methadone was implicated in DRD, but neither heroin nor buprenorphine was implicated,
ii) Heroin-specific DRDs: heroin was implicated in DRD, but neither methadone nor buprenorphine was implicated,
iii) DRDs in which methadone and heroin were both implicated, but buprenorphine was not.

In appraising which drugs are implicated as causal factors in any DRD and which, although present, probably did not contribute, Scotland’s pathologists are supported by having a national protocol for toxicological testing at forensic autopsies.

### 2.2 Scotland’s community health index and CHI-number

The CHI is a register of all patients in Scotland’s publicly funded healthcare system, NHS Scotland. Patients are identified by a 10-digit CHI-number, usually the patient’s date of birth (DDMMYY) followed by four digits: two randomly generated, the third identifying gender (odd for males) and the fourth a check digit (see Pavis and Morris (2015) on the criticality of CHI-numbers for Scotland’s efficient, trusted record-linkage).

### 2.3 Record-linkage for Scotland’s methadone-client cohort

To assess explanatory factors for methadone-specific DRDs among those who had been prescribed methadone in Scotland in 2009–2013, we needed to link methadone-prescriptions to NRS’s mortality records. Because all deaths are CHI-identified, Scotland’s CHI-number was used for this exact record-linkage. We defined Scotland’s methadone-prescription cohort as: clients who had received one or more CHI-identified methadone prescriptions (from GP or other-source) during 1 July, 2009–30 June 2013. In the Results section, below, we report the percentage of methadone prescriptions that were CHI-identified (63%) and the higher estimated percentage of methadone clients (82%) with at least one CHI-identified prescription.

The cohort was followed-up for mortality until 31 December 2013. Time at risk began at the date of the client’s first CHI-identified prescription during 1 July, 2009–30 June 2013 and ended at the earlier of death-date or 31 December 2013. The research-file was prepared in October 2014. As the requested date of the first CHI-identified prescription was missing for 61% of clients, the date of its re-imbursement was substituted. When both dates were provided, the re-imbursement date was, on average, 37 days later (sd 19 days).

Unlike in England and Wales, death registrations in Scotland occur within eight days of death having been ascertained (Bird, 2013, Bird, 2016); and NRS publishes official statistics on DRDs that occurred in year y in mid-August of the following year (y + 1).

Our record-linkage protocol was approved by Scotland’s Privacy Access Committee. To preserve client confidentiality, all CHI-identified methadone-prescriptions and death-records were pseudonymized in a manner which enabled the research team to link CHI-identified prescriptions and death-information for the same client.

### 2.4 Methadone-prescriptions

Methadone-prescriptions were accessed from Scotland’s Prescribing Information System (Alvarez-Madrazo et al., 2016) which contains information on all prescriptions written in Scotland and dispensed in the community. Only CHI-identified methadone-prescriptions for the same individual were linkable. The CHI-rate for methadone-prescriptions differed by year (higher in 2012/13 than in 2009/10) and prescription-source (higher for GPs than other-source prescriptions; see Dimitropoulou et al., 2016).

The quantity of methadone prescribed was always available as pharmacies are re-imbursed for the quantity prescribed. Duration of methadone-prescription and daily-dose could only be derived for GP prescriptions which had used an electronic dose instruction message. Natural language programming was applied to the free-text of this electronic message to abstract daily-dose (see Dimitropoulou et al. (2016) for the programme details). Extraction-rates for daily-dose of prescribed methadone were 79.3% from 140,361 electronic dose instruction GP-prescriptions in 2009/10 and 94.4% for 133,386 GP-prescriptions in 2012/13 (Dimitropoulou et al., 2016).

### 2.5 Statistical methods

Time at risk in days was converted to person-years (pys) by dividing the number of days at risk by 365.25 days. If prescription-date was missing, the later re-imbursement date was substituted (see above), which accounted for some negative time-intervals from the first CHI-identified prescription-date to death-date. Without loss of generality, we added 60 days to all times at risk and so fitted proportional hazards regression models to the time-at-risk plus 60 days, which avoided almost all negative times as the 90th percentile for the known re-imbursement delays was 60 days.

Only baseline covariates (that is: not time-varying, see below for reasons) were assessed, namely: those defined by the client’s first CHI-identified methadone-prescription between July 2009 and June 2013. Baseline covariates were prescription-source (GP vs. other-source); gender, age-group and their interaction; quintiles for the quantity of methadone prescribed; plus regions with historically high prevalence of heroin injecting (Greater Glasgow and Clyde, Lothian, Tayside, versus rest of Scotland) as a potential confounder.

Our reasons for not updating the quantity of methadone prescribed were three-fold: death informatively censors subsequent methadone-prescriptions; follow-up continued for six-months beyond the four years for accrual of CHI-identified methadone-prescriptions; and a client’s later methadone-prescriptions, even within the four years, need not be CHI-identified. Hence, the date of a client’s last CHI-identified methadone-prescription prior to 30 June 2013 does not necessarily indicate the date when methadone-prescribing ceased. By investigating accrual covariates only, which all clients have by definition, we introduced neither informative censoring nor ascertainment bias.

We investigated influential baseline covariates for methadone-specific DRDs, our focal interest; and for all DRDs, as the conventional analysis. As a sensitivity check, we analysed methadone-specific DRDs separately for clients accrued in 2009 (mostly prevalent) versus later (mostly incident).

For the subset of GP-clients for whom daily-dose of methadone at the first CHI-identified prescription was known, we also investigated quintiles for daily-dose of prescribed methadone.

We used STATA 12.1 for analysis. We present 95% confidence intervals for deaths per 1000 pys; and for hazard ratios (HRs).

## 3 Results

During 2009/10 to 2012/13, Scotland had 2,030,457 methadone prescriptions, 1,282,555 (63%) of which were CHI-identified. The CHI-identified prescriptions related to 33,128 methadone clients, 82% of Scotland’s 40,606 methadone clients during 2009/10 to 2012/13, as best-estimated using the method of Dimitropoulou et al. (2016).

The majority (63%, 20,758 clients) joined the cohort in July to December 2009, mostly prevalent clients. During follow-up of 121 254 pys to 31 December 2013, there were 1931 deaths. The cohort experienced 1171 non-DRDs and 760 DRDs at rates of 9.7 non-DRDs (95% CI: 9.1–10.2) and 6.3 DRDs (95% CIs: 5.8–6.7) per 1000 pys respectively. Buprenorphine was implicated in only seven DRDs and there were 67 DRDs in which none of heroin/morphine, methadone, or buprenorphine was implicated.

### 3.1 Demographic and other risk-factors

Methadone-specific DRDs (362) were more than double the cohort’s number of heroin-specific DRDs (173) or DRDs in which both heroin and methadone but not buprenorphine were implicated (151), see Table 1.

Opioid-specificity of clients’ DRDs varied by prescription-source, gender and age-group at cohort-entry. [The non-DRD: DRD ratio also varied by covariates, for example: higher for GP-prescribers and older clients.]

Clients whose first CHI-identified methadone-prescription was other-source had a higher methadone-specific DRD-rate. By gender, the male heroin-specific DRD-rate was significantly higher at 1.8 per 1,000 pys (5% CI: 1.4–2.0) than for females (0.8, 95% CI: 0.5–1.1) but clients’ methadone-specific DRD-rate was 3.0 irrespective of gender.

Clients whose age at first CHI-identified prescription in 2009–2013 was 35+ years had a DRD-rate of 7.7 (95% CI: 7.0–8.4) versus 4.9 (95% CI: 4.4–5.5) per 1,000 pys for younger clients. In particular, methadone-specific DRD-rates were substantially higher in the older age-group than for younger clients: 4.2 (95% CI: 3.6–4.7) versus 1.9 (95% CI: 1.5–2.2) per 1,000 pys.

For older clients, neither the DRD-rate nor the methadone-specific DRD-rate was lower for females. By contrast, in the younger age-group, the DRD-rate was higher for males than females, see Table 1: interaction.

Regional variation in the methadone-specific DRD-rates was unremarkable (chi-square on 3df of 5.78, p = 0.12). However, Tayside’s significantly high DRD-rate could be a confounder when analysing DRDs.

### 3.2 Proportional hazards analyses

Only four time-intervals remained negative after the universal addition of 60 days (see Methods section) and so, without loss of generality, these few clients were excluded from regression analyses. Two were DRDs, one of them methadone-specific.

Quintiles for the quantity of methadone prescribed at first CHI-identified prescription were as follows: < = 280; 281–700; 701–1200; 1201–1960; and >1960 mg. As shown in Supplementary material^2^, quintiles for daily dose of prescribed methadone in the GP-subset with e-readable dose instructions were: < = 30; 31–50; 51–70; 71–90; and >90 mg.

With neither region nor gender by age-group interaction fitted and irrespective of whether quintiles were fitted, clients’ age-related HRs were steeper for methadone-specific DRDs (Table 2, top panel) than for all DRDs (Table 3, top panel): for example, HRs of 1.9 and 2.9 for the two oldest age-group in Table 2 versus 1.4 and 1.9 in Table 3. The highest quintile for quantity of prescribed methadone was associated with a HR for 1.8 (95% CI: 1.3–2.5) for methadone-specific DRDs but not for all DRDs.

The lower panel of Table 3 confirmed the familiar gender by age-group interaction for DRD-hazards, and showed that confounding by region was largely responsible for the apparently higher DRD-hazard in Table 1 for other-source prescriptions.

There was no female advantage when it came to methadone-specific DRDs, nor regional differences (unlike for all DRDs). For clients whose first CHI-identified prescription was other-source, the HR for methadone-specific DRDs was 1.4 (95% CI: 1.2–1.8).

For mostly prevalent clients (those recruited in July to December 2009), the quantity of prescribed methadone at cohort-entry was likely to represent the clients’ typical or stabilized prescription but, for mostly incident clients (those recruited after 2009, for 34% of whom the quantity of prescribed methadone was in the lowest quintile), quantity prescribed at cohort-entry was more likely to represent initial prescribing. See Supplementary material^3^ for pH analysis of methadone-specific DRDs separately for clients who accrued in July to December 2009 (mostly prevalent) versus later. The stratification was highly significant (chi-square test on 9 df of 28.21, p ∼0.0009) with four main points of difference. Other-source prescribing applied for half the mostly incident clients (versus only 22% of 2009-accrued clients) and, for them, was associated with a significantly greater hazard of methadone-specific DRD (test for interaction: p ∼0.05). For clients aged 45+ years, the HR for methadone-specific DRD was more extreme for mostly incident than mostly prevalent clients. Thirdly, clients who entered the cohort after 2009 with a baseline quantity of prescribed methadone of 281–700 mg were at relatively greater risk of methadone-specific DRD than their mainly prevalent counterparts. Fourth, the highest quintile for quantity of prescribed methadone applied to only 13% of mostly incident clients but was associated with a notably high HR of 2.8 (95% CI: 1.5–5.1) for methadone-specific DRD.

For the GP-subset of clients with known daily-dose at their first CHI-identified prescription, the highest quintile for daily-dose of prescribed methadone (>90 mg) may be even more prognostic than for quantity prescribed, see Supplementary material^4^

## 4 Discussion

### 4.1 Summary of main findings

Scotland’s methadone-prescription cohort reveals for the first time that clients’ age-related hazard increases more steeply for methadone-specific DRDs than for all DRDs (e.g.,HR for males aged 45+ years: 2.9 versus 1.9). Although Pierce et al., 2015a, Pierce et al., 2016 showed that DRD-rates increased beyond 45 years of age for England’s drug treatment clients, they did not have information on the opioid-specificity of DRDs to enable an analysis of methadone-specific DRDs. Nor did they have information on the quantity of prescribed methadone. Importantly, the steeply age-related HRs for methadone-specific DRDs in Scotland’s methadone client cohort were evident in Table 2, even without fitting quintiles for the quantity of methadone and so validation studies in other OST cohorts, which are necessary, can proceed primarily by ascertaining the opioid-specificity of their DRDs.

Unlike for all DRDs, there was no overall hazard-reduction for females in respect of methadone-specific DRDs; no gender by age-group interaction, see Table 1; and consistent with the Scottish Drug Strategy Delivery Commission’s endorsement of the professional standards by which OST was delivered in Scotland, no regional variation. For methadone-specific DRDs, but not all DRDs, the highest quintile for quantity of prescribed methadone was associated with significantly increased hazard (1.8; 95% CI: 1.3–2.5).

### 4.2 Key considerations

Unlike buprenorphine, methadone − alone and with other drugs commonly used for mental health conditions (see Medicines and Healthcare products Regulatoy Agency (MHRA), 2006) and NHS Greater Glasgow and Clyde Medicines Information Service, 2012)—is known to affect the electrical conductivity of the heart muscle. Specifically, prolongation of part of the normal electrical cycle, known as the QT interval, and measurable by an electrocardiogram (ECG), can occur (Wedman et al., 2007, Kornick et al., 2003, Peles et al., 2007, Weimer and Chou, 2014, Chou et al., 2014a, Chou et al., 2014b, Bohnert et al., 2001, Gomes et al., 2011, Liang and Yurner, 2015). Prolongation of the corrected QT interval (cQT) and its sequelae of torsades de pointes has been associated with the risk of sudden cardiac death (Maremmani et al., 2005, Roden, 2006, Justo et al., 2006, Fanoe et al., 2007, Chugh et al., 2008). Whether undiagnosed QTc prolongation contributes to the tally of methadone-specific DRDs is unknowable because prolongation leaves no detectable trace at autopsy (Chugh et al., 2008). Other risk factors for QTc prolongation include co-morbidities such as renal impairment, heart or liver disease; and being female (Makkar et al., 1993, Kest et al., 2000, Lee and Ho, 2013, Graziani and Nistico, 2015, Bawor et al., 2014).

In 2006, MHRA recommended that any patient requiring more than 100 mg of methadone per day should be closely monitored. Periodic heart monitoring of methadone clients has been recommended to detect QTc prolongation by 60 milliseconds or to above 500 milliseconds, but evidence on the effectiveness of such screening is weak (Pani et al., 2013): with detection in perhaps 2% of clients and mostly on doses greater than 100 mg daily. The quantity of prescribed methadone was >1960 mg for a fifth of Scotland’s methadone-prescription clients (and >90 mg daily for 18% of the GP subset) and so we speculate that the ECG-detection rate for QTc prolongation could between 5% and 10% for Scotland’s methadone-prescription clients in the top quintile of prescribed methadone. Moreover, ECGs on a representative sample of only 200 clients from the top quintile, if they were willing to attend for screening, could estimate the detection-rate for QTc prolongation rather well: with a standard error of about 2%. However, the clear HR warning in Table 2 is that precautionary monitoring, including by ECG, may be warranted for all methadone clients over 45 years of age.

In view of the above risk-factors for QTc prolongation, future record-linkage for Scotland’s CHI-identified methadone clients will seek to link-in not only clients’ diagnoses for blood-borne viruses (Hepatitis B, C and HIV) but also cardiac, gastro-intestinal, alcohol-related (Hutchinson et al., 2005, McDonald et al., 2008, McDonald et al., 2010) and mental-health hospitalizations to try to unravel contributory factors to methadone-specific DRDs’ strong age-gradient and absent gender effect. Co-prescription of benzodiazepines is likely to be of little help, however, as illicit access predominates at DRDs (Barnsdale et al., 2015, Barnsdale et al., 2016). Self-reported misuse of benzodiazepines has, however, been shown to increase DRD-risk (Pierce et al., 2016).

The quantity of methadone prescribed at cohort-entry was available for all clients. Quantity prescribed played a key role in clients’ risk of methadone-specific DRD, but not for all DRDs. For the GP subset, daily-dose of prescribed methadone (>90 mg) underlined the association. Despite inevitable uncertainties about causation (Hill, 1965), the novel findings from Scotland—a first and so requiring external corroboration—serve to emphasise 21st century alerts to prescribers and their opioid-dependent clients, especially new clients, to be cautious if *either* the quantity of methadone prescribed exceeds 1,960 mg (for example: 140 mg for 14 days; or, typically, over 90 mg daily for 28 days but dispensed in 21 instalments, see Supplementary material^5^); *or* daily-dose exceeds 90 mg. Similar warnings about daily-dose in excess of 100 mg have been enunciated for patients receiving methadone for non-cancer pain (Chou et al., 2014a, Chou et al., 2014b) who, for the most part, would not have progressive liver disease (Gibson et al., 2011, Pierce et al., 2015a) or other co-morbidities. Co-morbidities clearly affect Scotland’s ageing methadone clients (Hutchinson et al., 2005, McDonald et al., 2008, McDonald et al., 2010, Kimber et al., 2010, Merrall et al., 2012). The Scottish data suggests that prescribers may need to discuss risk-mitigation with all methadone clients over 45 years of age.

We found an increased adjusted hazard of methadone-specific DRD for other-source prescribers, more particularly for incident clients. A contributory factor here could be that the physical and psychiatric history, and hence frailty of these clients, is less well-known to other-source prescribers than by GPs.

### 4.3 Strengths and limitations of this study

First, Scotland has a national protocol for toxicological testing at forensic autopsy which underpins the opioid-specificity of its DRDs. Secondly, unparalleled for a national cohort (Sullivan et al., 2014), we could analyse the quantity of methadone prescribed at cohort-entry in addition to demographic risks for a large cohort of 33,000 methadone-clients who experienced 760 recent-past DRDs, 362 of them methadone-specific DRDs.

Third, to minimize ascertainment bias, we considered only the baseline (not time-varying) quantity of methadone prescribed at the first CHI-identified methadone prescription. This first CHI-identified prescription defined the client’s entry to Scotland’s methadone-client cohort (2009–2013) but was typically not the client’s first methadone prescription, especially if cohort-entry was in 2009. We therefore investigated covariate influences on methadone-specific DRDs separately for mostly prevalent clients versus those accrued after 2009 whose baseline quantity of prescribed methadone may have been pre-stabilization. Besides a differential association with other-source prescribing, we found that two other hazards for methadone-specific DRD were more extreme for the mostly incident than for mostly prevalent clients: aged 45+ years and highest quintile for quantity prescribed. Both underscore our main findings.

Turning to limitations, of which there are several, we acknowledge first that only 63% of all methadone prescriptions during 2009/10 to 2012/13 were CHI-identified. However, our 33,128 CHI-identified methadone clients represented, at a best estimate, 82% of Scotland’s methadone-clients over the four years (plausible range: 70% to 93%), a more critical measure of completeness being client-based.

The unavailability of daily-dose for other-source prescriptions and for half of the first CHI-identified prescriptions by GPs is, of course, regrettable: and especially so as our analysis in Supplementary material^6^ for the GP-subset suggested that the highest quintile for daily prescribed methadone was more prognostic even than the quantity prescribed. Irrespective of whether daily-dose was available or not, summary statistics on the number of prescription-instalments by GPs were reassuringly similar so that any bias in the GP-subset may be more apparent than real. Nonetheless, we’d have preferred that daily-dose at first CHI-identified prescription had been accessible electronically for 80% or more clients.

The need to substitute, for missing first prescription-date, the later reimbursement-date was a minor issue. More importantly, we did not know, and hence could not analyse (Cornish et al., 2010, Pierce et al., 2015a, Cousins et al., 2016), when clients exited from methadone therapy as the date of their last CHI-identified methadone-prescription does not exclude later non-CHI prescriptions. Hence, once included in the cohort, clients have remained in follow-up.

A fifth limitation, in common with most unconsented record-linkage studies, is that we had little scope for resolving data-queries, such as some apparent inconsistencies (2%) between quantity of methadone prescribed and daily-dose.

Benzodiazepines (licit or illicit), alcohol and methadone conspire in terms of DRD-risk (Merrall et al., 2012, Pierce et al., 2015a, Pierce et al., 2016), as evidenced by their co-presence at DRDs (Bird et al., 2010, Bird and Robertson, 2011, Barnsdale et al., 2015, Barnsdale et al., 2016) and by the co-prescription of benzodiazepines with opioids for pain (Christie, 2011, Leece et al., 2015, Park et al., 2015). We did not request that methadone clients’ co-prescriptions for benzodiazepines be linked-in because the added time and complexity would not have been warranted given that illicit supplies would remain unaccounted for and, among Scotland’s DRDs, are the substantial majority (Barnsdale et al., 2015, Barnsdale et al., 2016).

Seventh, association is not causation (Hill, 1965): age over 45 years and higher quantity of methadone prescribed may be respectively demographic and pharmacological markers for harder-to-support clients whose opioid dependency is chronic and likewise their HCV progression (Eap et al., 2002). Research into gender differences in opioid analgesia and addiction has given conflicting results (Lee and Ho, 2013, Graziani and Nistico, 2015, Bawor et al., 2014) but being female is a risk-factor for QTc prolongation (Makkar et al., 1993), which may partly explain why, for females, the risk of methadone-specific DRDs is *not l*ower than for their male counterparts.

## 5 Conclusions

Vindicating our hypothesis, Scotland’s methadone-prescription cohort has revealed high methadone-specific DRD rates in older clients irrespective of gender; and increased hazard for the top quintile of prescribed methadone. Better understanding is needed of the pharmaco-dynamics of methadone for older, opioid-dependent clients, many with progressive liver, cardiovascular or other disease.

Guidelines on the prescribing of methadone in the UK will be updated in 2016. The Scottish data signal that methadone-prescribers and other specialists should exercise particular caution in managing older clients, many of whom are HCV-infected or alcohol-dependent; and if their daily-dose of methadone exceeds 90 mg or the quantity of methadone per prescription exceeds 1960 mg.

Higher methadone-specific DRD-rate for older clients, and the absence of a gender differential, should be noted by prescribers and by clients themselves. We recommend that take-home naloxone be prescribed for methadone-clients (Strang et al., 2014, Bird et al., 2015, Bird et al., 2016), especially those aged 35+ years.

## Funding

SMB & PD and the record-linkages were funded by Medical Research Council programme number U105260794. LG is funded by the Medical Research Council programme number U105292687.

## Role of funder

The funder had no role in the writing of, or decision to publish, this paper.

## Data sharing

All data presented were accessed by us, and are accessible by others, only by agreement of Scotland’s Privacy Access Committee.

## Ethical approvals

Approval in the public interest was obtained from Scotland’s Privacy Access Committee for analysis of Scotland’s methadone prescriptions spanning four years from 2009/10 to 2012/13, taking account not only of region, gender and age-group but quantity prescribed in relation to the hazard of DRDs, and of methadone-specific DRDs. The research team received only anonymized data from which deductive disclosure about individual clients could not be made.

## Contributions

SMB & JRR hypothesised that methadone prescribing might be implicated in the weakening of the female advantage in terms of drugs-related deaths as opioid-users aged.

SMB, SMcT and MB submitted an application to Scotland’s Privacy Access Committee for CHI-based linkage of Scotland’s methadone prescriptions in July 2009 to June 2013 and survival-status to 31 December 2013. Deaths were notified DRDs or non-DRDs and, for each DRD, whether three specific opioids (heroin, methadone, buprenorphine) were implicated in order to analyse the role of methadone prescribing in the opioid-specificity of Scotland’s DRDs.

PD, supervised by SMB, merged the pseudonymized data-files, analysed the persistence of GP-clients across prescription-years, defined the time-at-risk for Scotland’s CHI-identified methadone clients, and conducted the exploratory life-table analyses.

LG conducted the proportional hazards analyses.

SMB and JRR initially drafted the paper with editing by all co-authors, MB and SMcT especially.

## Conflicts of interest

SMB holds GSK shares. SMB is co-principal investigator for the MRC-funded prison-based N-ALIVE pilot Trial. SMB served on Scotland’s National Naloxone Advisory Group (2010–2016). SMB was a co-grant-holder for MRC-funded addictions cluster, NIQUAD (Nationally Integrated Quantitative Assessment of Drug Harms).

JRR chaired Scotland’s National Forum on Drugs-Related Deaths and currently chairs Scotland’s Harm Reduction Panel; and serves on the sub-group which is updating UK’s guidelines on methadone prescribing.

LG and PD have no conflicts of interest.

SMcT and MB have no conflicts of interest.

## Figures and Tables

**Table 1:** For major sub-groups, numbers of deaths by defined opioid-specificity and person-years (pys) at risk since first CHI-identified prescription to earlier of death-date or 31 December 2013.

**Sub-group defined by 1^st^ CHI-identified prescription**	**Clients**	**Non-DRDs**	**DRDs**	**Opioid-specific DRD sub-groups**			****Person-years, pys****
				Heroin: yes	Heroin: no	Heroin: yes	
				Meth: no	Meth: yes	Meth: yes	
				Bupren: no	Bupren: no	Bupren: no	
Scotland-wide	**33 128**	**1171**	**760**	173	362	151	**121 254 pys**
Death-rate per		**9.7**	**6.3**	**1.4**	**3.0**	**1.2**	
1000 pys (95% CI)		(9.1–10.2)	(5.8–6.7)	(1.2–1.6)	(2.7–3.3)	(1.0–1.4)	
***Prescription source***							
GP-prescription	**22 114**	**887**	**502**	130	232	86	**84 338 pys**
Death-rate per		**10.5**	**6.0**	**1.5**	**2.8**	**1.0**	
1000 pys (95% CI)		(9.8–11.2)	(5.4–6.5)	(1.3–1.8)	(2.4–3.1)	(0.8–1.2)	
Other-source	****11 014****	**284**	**258**	43	130	65	**36 916 pys**
Death-rate per		**7.7**	**7.0**	**1.2**	**3.5**	**1.8**	
1000 pys (95% CI)		(6.8–8.6)	(6.1–7.8)	(0.8–1.5)	(2.9–4.1)	(1.3–2.2)	
***Gender***							
Males	**22 025**	**827**	**535**	141	240	108	**80 394 pys**
Death-rate per		**10.3**	**6.7**	**1.8**	**3.0**	**1.3**	
1000 pys (95% CI)		(9.6–11.0)	(6.1–7.2)	(1.4–2.0)	(2.6–3.4)	(1.1–1.6)	
Females	**11 103**	**344**	**225**	32	122	43	**40 860 pys**
Death-rate per		**8.4**	**5.5**	**0.8**	**3.0**	**1.1**	
1000 pys (95% CI)		(7.5–9.3)	(4.8–6.2)	(0.5–1.1)	(2.4–3.5)	(2.4–3.5)	
***Age-group at Cohort-Entry***							
35+ years @ Cohort-entry	**16 002**	**886**	**451**	78	244	86	**58 462 pys**
Death-rate per		**15.2**	**7.7**	**1.3**	**4.2**	**1.5**	
1000 pys (95% CI)		(14.2–16.2)	(7.0–8.4)	(1.0–1.6)	(3.6–4.7)	(1.2–1.8)	
< 35 years @ Cohort-entry	**17 126**	**285**	**309**	95	118	65	**62 792 pys**
Death-rate per		**4.5**	**4.9**	**1.5**	**1.9**	**1.0**	
1000 pys (95% CI)		(4.0–5.1)	(4.4–5.5)	(1.2–1.8)	(1.5–2.2)	(0.8–1.3)	
***Gender by Age-group at Cohort-Entry: interaction***							
Male, 35+	**11 540**	**632**	**320**	68	163	63	**42 126 pys**
Death-rate per		**15.0**	**7.6**	**1.6**	**3.9**	**1.5**	
1000 pys (95% CI)		(13.8–16.2)	(6.8–8.4)	(1.2–2.0)	(3.3–4.5)	(1.1–1.9)	
Female, 35+	**4 462**	**254**	**131**	10	81	23	**16 336 pys**
Death-rate per		**15.5**	**8.0**	**0.6**	**5.0**	**1.4**	
1000 pys (95% CI)		(13.6–17.5)	(6.6–9.4)	(0.3–1.1)	(3.9–6.0)	(0.9–2.1)	
Male, < 35	**10 485**	**195**	**215**	73	77	45	**38 268 pys**
Death-rate per		**5.1**	**5.6**	**1.9**	**2.0**	**1.0**	
1000 pys (95% CI)		(4.4–5.8)	(4.8–6.4)	(1.5–2.4)	(1.5–2.5)	(0.8–1.5)	
Female, < 35	**6 641**	**90**	**94**	22	41	20	**24 524 pys**
Death-rate per		**3.7**	**3.8**	**0.9**	**1.7**	**0.8**	
1000 pys (95% CI)		(2.9–4.4)	(3.1–4.6)	(0.5–1.4)	(1.1–2.2)	(0.5–1.3)	
***Region at cohort entry***							
G. Glasgow & Clyde	**12 261**	**525**	**307**	82	135	56	**47 465 pys**
Death-rate per		**11.1**	**6.5**	**1.7**	**2.8**	**1.2**	
1000 pys (95% CI)		(10.2–12.0)	(5.8–7.2)	(1.4–2.1)	(2.6–4.3)	(0.9–1.5)	
Lothian	**4 801**	**170**	**84**	10	58	11	**17 423 pys**
Death-rate per		**9.8**	**4.8**	**0.6**	**3.3**	**0.6**	
1000 pys (95% CI)		(8.4–11.3)	(3.9–6.0)	(0.3–1.1)	(2.5–4.2)	(0.4–1.1)	
Tayside	**2 760**	**87**	**74**	7	34	23	**8 034 pys**
Death-rate per		**10.8**	**9.2**	**0.9**	**4.2**	**2.9**	
1000 pys (95% CI)		(8.8–13.4)	(7.3–11.6)	(0.4–1.8)	(3.0–5.9)	(1.9–4.3)	
Rest of Scotland	**13 306**	**389**	**295**	74	135	61	**48 335 pys**
Death-rate per		**8.0**	**6.1**	**1.5**	**2.8**	**1.3**	
1000 pys (95% CI)		(7.2–8.8)	(5.4–6.8)	(1.2–1.9)	(2.4–3.3)	(1.0–1.6)	

**Table 2:** Proportional hazards analysis from first CHI-identified prescription to methadone-specific DRDs: with adjustment for baseline covariates excluding or including quintiles for prescribed quantity of methadone.

Covariates	361 Methadone-specific DRDs; 33,124 at-risk clients Regression chi-square test on 3df for interaction (gender*age-group) when region also fitted = 3.30, p ∼0.35					
	Excluding QUINTILES for prescribed quantity			Including QUINTILES for prescribed quantity Regression chi-square (9 df) = 98.88		
	HR	95% CI	p-value	HR	95% CI	p-value
No interaction needed for gender * age-group						
Regression chi-square (4df) for QUINTILES				22.98, p ∼0.0001		
Other-source	1.42	1.15–1.77	0.001	1.44	1.16–1.79	0.001
Female	1.12	0.90–−1.39	0.322	1.14	0.91–1.42	0.246
AGE-GROUP with 25–34 years at first CHI-identified prescription as baseline						
Age < 25	0.51	0.26–1.00	0.050	0.53	0.27–1.04	0.064
Age 35–44	1.95	1.53–2.48	<0.001	1.91	1.50–2.44	<0.001
Aged 45+	2.95	2.18–4.00	<0.001	2.90	2.14–3.93	<0.001
QUINTILES for prescribed quantity of methadone with lowest quintile as baseline						
Quintile 2: 281–700				1.10	0.76–1.59	0.604
Quintile 3: 701–1200				0.90	0.61–1.32	0.577
Quintile 4: 1201–1960				1.22	0.86–1.74	0.261
Quintile 5: > 1960 mg (For example, 1960 mg = 14 days@140 mg daily or 28 days @ 70 mg)				1.79	1.29–2.50	<0.001
When covariates for REGION [Greater Glasgow and Clyde (as baseline) versus Lothian, Tayside, rest of Scotland] were added to the above models, neither regression chi-square (on 3df) for REGION was statistically significant, see below.						
Regression chi-square (3df) for REGION	5.80, p ∼0.12			2.42, p ∼0.49		

**Table 3:** Proportional hazards analysis from first CHI-identified prescription to DRD: with adjustment for baseline covariates excluding or including quintiles for prescribed quantity of methadone.




## Footnotes

1 Supplementary material can be found by accessing the online version of this paper at http://dx.doi.org and by entering doi:…

2 Supplementary material can be found by accessing the online version of this paper at http://dx.doi.org and by entering doi:…

3 Supplementary material can be found by accessing the online version of this paper at http://dx.doi.org and by entering doi:…

4 Supplementary material can be found by accessing the online version of this paper at http://dx.doi.org and by entering doi:…

5 Supplementary material can be found by accessing the online version of this paper at http://dx.doi.org and by entering doi:…

6 Supplementary material can be found by accessing the online version of this paper at http://dx.doi.org and by entering doi:…
