# Supplementary material for: Risk-factors for methadone-specific deaths in Scotland’s methadone-prescription clients between 2009 and 2013*
Source: Drug Alcohol Depend. 2016 Oct 1;167:214–23. doi: 10.1016/j.drugalcdep.2016.08.627 (PMC5047032; doi:10.1016/j.drugalcdep.2016.08.627)
Supplement: Supplementary file 1 [file mmc1.docx]

***Supplementary Material for the article:***

**Risk-factors for methadone-specific deaths in Scotland’s methadone-prescription clients between 2009 and 2013**

*Lu Gao^1^, Polyxeni Dimitropoulou^1^, J. Roy Robertson^2^, Stuart McTaggart^3^, Marion Bennie^34^,*

*Sheila M. Bird^15^*

^1^MRC Biostatistics Unit, CAMBRIDGE CB2 0SR

^2^Usher Institute of Population Health Sciences and Informatics, Edinburgh University, EDINBURGH EH16 4UX

^3^Information Services Division, NHS National Services Scotland, EDINBURGH EH12 9EB

^4^Strathclyde Institute of Pharmacy and Biomedical Sciences, University of Strathclyde,

GLASGOW G4 0RE

^5^Department of Mathematics and Statistics, Strathclyde University, GLASGOW G1 1XH.

Corresponding author: Sheila M. Bird ([sheila.bird@mrc-bsu.cam.ac.uk](mailto:sheila.bird@mrc-bsu.cam.ac.uk))

***This material supplements, but does not replace, the peer-reviewed article in***

***Drug and Alcohol Dependence.***

*Persistence of GPs’ methadone-clients*

Using only the CHI-identified GP prescriptions from the merged file of methadone prescriptions, we defined three era-pairs with a 1-year span (namely: 2009/10 and 2010/11; or 2010/11 and 2011/12; or 2011/12 and 2012/13); two era-pairs with a 2-year span (namely: 2009/10 and 2011/12; or 2010/11 and 2012/13); or one era-pair with a 3-year span (2009/10 and 2012/13). We then counted how many of the CHI-identified GP methadone-clients, who featured in one or other era, had CHI-identified methadone prescriptions in both eras. Finally, we also documented how many of the CHI-identified GP methadone-clients who had a CHI-identified prescription in any of the four eras actually had CHI-identified GP-prescriptions in all four. Persistence of a client’s CHI across eras does not mean that the client was persistently in methadone treatment throughout each era.

**Table S1** shows that the proportion of methadone-prescriptions by GPs in Scotland that were CHI-identified increased from 78% in 2009/10 to 86% in 2012/13 but that GPs’ number of CHI-identified methadone-prescriptions reduced from 247,531 to 229,204. The number of GP-clients with one or more CHI-identified methadone-prescription also reduced from 18,085 in 2009/10 to 16,905 in 2012/13. The median and upper 90^th^ percentile for GPs’ number of CHI-identified methadone-prescription per client were stable at 13 and 24 respectively.

**Table S1.** GP methadone-prescriptions by era, whether CHI-identified and CHI-identified methadone client numbers.

| Era | GP-prescriptions | CHI-identified GP-prescriptions | | | | |
| --- | --- | --- | --- | --- | --- | --- |
|  |  | Number of  CHI-identified scripts  (% GP-total) | Total number of clients with one or more CHI-identified scripts | Clients with single CHI-identified script | Median number of CHI-identified scripts per client | Upper 90^th^ percentile for CHI-identified scripts per client |
| 2009/10 | 316157 | 247531 (78.3%) | 18,085 | 648 | 13 | 24 |
| 2010/11 | 314497 | 255488 (81.2%) | 18,361 | 585 | 13 | 24 |
| 2011/12 | 293250 | 248057 (84.6%) | 17,780 | 580 | 13 | 24 |
| 2012/13 | 265957 | 229204 (86.2%) | 16,905 | 528 | 13 | 23 |

As shown in **Table S2**, persistence as a CHI-identified GP methadone-client was 78% across eras with a 1-year span, 66% across eras with a 2-year span and 59% across a 3-year span. There were 23,336 clients with one or more CHI-identified GP-prescription in the combined 4-year database, of whom 12,248 (52.5%) had CHI-identified GP-prescriptions for methadone in each of the four eras.

**Table S2.** CHI-identified GP prescriptions for methadone by era-pairs and across all four eras.

| Era-pair | Number of clients with one or more  CHI-identified GP prescription | Number of clients with one or more  CHI-identified GP prescriptions in the combined file | Number of clients in combined file with  CHI-identified GP-prescriptions in single file only; **or in both/all eras (%)** |
| --- | --- | --- | --- |
| 2009/10 with | 18,085 |  | 2,111 |
| 2010/11: 1-year span | 18,361 |  | 2,387 |
| ***ACROSS BOTH ERAS*** |  | 20,472 | **15,974 (78.0%)** |
| 2010/11 with | 18,361 |  | 2,538 |
| 2011/12: 1-year span | 17,780 |  | 1,957 |
| ***ACROSS BOTH ERAS*** |  | 20,318 | **15,823 (77.9%)** |
| 2011/12 with | 17,780 |  | 2,636 |
| 2012/13: 1-year span | 16,905 |  | 1,761 |
| ***ACROSS BOTH ERAS*** |  | 19,541 | **15,144 (77.5%)** |
|  | | | |
| 2009/10 with | 18,085 |  | 3,722 |
| 2011/12: 2-year span | 17,780 |  | 3,417 |
| ***ACROSS BOTH ERAS*** |  | 21,502 | **14,363 (66.8%)** |
| 2010/11 with | 18,361 |  | 4,360 |
| 2012/13: 2-year span | 16,905 |  | 2,904 |
| ***ACROSS BOTH ERAS*** |  | 21,265 | **14,001 (65.8%)** |
|  | | | |
| 2009/10 with | 18,085 |  | 5,139 |
| 2012/13: 3-year span | 16,905 |  | 3,959 |
| ***ACROSS BOTH ERAS*** |  | 22,044 | **12,946 (58.7%)** |
|  | | | |
| 2009/10 with | 18,085 |  | 1,599 |
| 2010/11 with | 18,361 |  | 510 |
| 2011/12 with | 17,780 |  | 417 |
| 2012/13 | 16,905 |  | 1,253 |
| ***ACROSS ALL 4-ERAS*** |  | 23,336 | **12,248 (52.5%)** |

*Stratification: by whether CHI-identified methadone client accrued in July to December 2009 or later.*

**All clients:** Per quintile before restriction to the GP-subset (see **Table S3**), the mean number of instalments for prevalent versus incident clients was very significantly different between the 2009-accrued versus incident clients: typically higher for incident clients.

As the quantity prescribed increased, so too did the mean number of instalments in which the prescriptions was issued: for incident clients, the mean was four instalments per prescription in the lowest quintile but 23 instalments in the highest quintile for quantity prescribed.

Over half (6,729) of the 12,370 incident clients had a baseline quantity of prescribed methadone in the lower two quintiles.

**GP-subset:** Per quintile, the mean number of instalments differed immaterially between 2009-accrued and incident GP-clients. Over two-thirds (4,097) of the 5,950 incident clients in the GP-subset had a baseline quantity of prescribed methadone in the lower two quintiles.

The baseline mean daily-dose prescribed by GPs was very significantly higher for incident than for prevalent clients in the three highest quintiles. For example, in the highest quintile, the mean daily-dose was 98.7 mg for incident clients versus 92.8 mg for those who joined the methadone cohort in July to December 2009.

In the GP-subset, daily-dose was known for 2209/3716 (59%) prevalent clients and 466/627 (74%) incident clients in the highest quintile for quantity prescribed, in the lowest quintile for quantity, the daily-dose was available for only 37% of clients (prevalent, 939/2537; or incident, 1081/2890).

**Table S3.** Controlling for quantity of prescribed methadone, comparison between methadone clients accrued in July to December 2009 versus later (mostly incident clients) in respect of: mean (se) for number of instalments and, for the GP-subset, mean (se) for daily-dose of methadone.

| QUINTILE for QUANTITY | Subset | Comparator | Methadone clients  accrued to cohort in 2009 | | Methadone clients accrued after 2009 | |
| --- | --- | --- | --- | --- | --- | --- |
|  |  |  | Mean (se) | Number of clients | Mean (se) | Number of clients |
| Q1:  <=280 mg | **All ***** | **Instalments** | **4.45 (0.082)** | **3025** | **4.13 (0.052)** | **4229** |
|  | GP-subset | Instalments | 4.42 (0.090) | 2537 | 4.21 (0.059) | 2890 |
|  | GP-subset | *Daily-dose* | *35.3 mg (0.82)* | 939 | *34.0 mg (0.60)* | 1081 |
| Q2:  281- 700 | **All ***** | **Instalments** | **8.38 (0.100)** | **4155** | **9.26 (0.118)** | **2500** |
|  | GP-subset | Instalments | 8.27 (0.117) | 3260 | 7.97 (0.027) | 1207 |
|  | GP-subset | *Daily-dose* | *46.3 mg (0.77)* | 1280 | *47.1 mg (1.14)* | 547 |
| Q3:  701-1200 | **All ***** | **Instalments** | **11.81 (0.118)** | **4198** | **15.13 (0.197)** | **1803** |
|  | GP-subset | Instalments | 12.23 (0.147) | 3052 | 12.68 (0.330) | 607 |
|  | GP-subset | *Daily-dose*** | *56.1 mg (0.70)* | 1285 | *59.8 mg (1.48)* | 344 |
| Q4:  1201-1960 | **All ***** | **Instalments** | **15.10 (0.137)** | **4734** | **19.90 (0.195)** | **2213** |
|  | GP-subset | Instalments | 15.43 (0.159) | 3599 | 15.28 (0.377) | 619 |
|  | GP-subset | *Daily-dose**** | *67.2 mg (0.56)* | 1811 | *72.5 mg (1.37)* | 418 |
| Q5:  > 1960 mg | **All ***** | **Instalments** | **20.97 (0.176)** | **4646** | **22.78 (0.256)** | **1625** |
|  | GP-subset | Instalments | 21.01 (0.205) | 3716 | 21.45 (0.478) | 627 |
|  | GP-subset | *Daily-dose**** | *92.8 mg (0.69)* | 2209 | *98.7 mg (1.32)* | 466 |

*** denotes P < 0.001 for comparison of 2009 versus later accrual

*Separately for clients who accrued in July to December 2009 (mostly prevalent) versus later: PH analysis of methadone-specific DRDs.*

The above stratification was highly significant (chi-square test on 9 df of 28.21, p ~ 0.0009) with four main points of difference, see **Table S4**. Other-source prescribing applied for half the mostly incident clients (versus only 22% of 2009-accrued clients) and, for them, was associated with a significantly greater hazard of methadone-specific DRD (test for interaction: p ~ 0.05). For clients aged 45+ years, the HR for methadone-specific DRD was more extreme for mostly incident than prevalent clients. Thirdly, clients who entered the cohort after 2009 with a baseline quantity of prescribed methadone of 281 to 700mg were at relatively greater risk of methadone-specific DRD than their mainly prevalent counterparts. Fourth, the highest quintile for quantity of prescribed methadone applied to only 13% of mostly incident clients but was associated with a notably high HR of 2.8 (95% CI: 1.5 to 5.1) for methadone-specific DRD.

**Table S4.** Proportional hazards analysis from first CHI-identified prescription to methadone-specific DRD: separately for clients who entered the cohort in July to December 2009 (mainly prevalent) or after 2009.

| Covariate | **263 Methadone-specific DRDs**;  20 757 at-risk clients  ***Regression chi-square (9 df) = 56.13*** | | | | **98 Methadone-specific DRDs**;  12 367 at-risk clients  ***Regression chi-square (9 df) = 70.96*** | | | |
| --- | --- | --- | --- | --- | --- | --- | --- | --- |
|  | **Number of clients** | HR | 95% CI | p-value | HR | 95% CI | p-value | **Number of clients** |
| Regression contribution by QUINTILES | Chi-square on 4df = 14.63, p~0.0055 | | | | Chi-square on 4df = 23.32, p ~0.00011 | | | |
| Other-source | **4 594** | 1.18 | 0.88-1.58 | 0.259 | 2.07 | 1.32-3.25 | 0.002 | **6 417** |
| Female | **6 999** | 1.18 | 0.91-1.52 | 0.204 | 1.05 | 0.68-1.62 | 0.814 | **4 104** |
| AGE-GROUP with 25-34 years at first CHI-identified prescription as baseline | | | | | | | | |
| Age < 25 | **1 054** | 0.31 | 0.10-1.00 | 0.050 | 0.85 | 0.35-2.06 | 0.722 | **1 455** |
| Age 35-44 | **8 395** | 1.86 | 1.41-2.45 | <0.001 | 2.01 | 1.23-3.29 | 0.005 | **3 982** |
| Aged 45+ | **2 223** | 2.24 | 1.54-3.27 | <0.001 | 5.21 | 3.06-8.87 | <0.001 | **1 399** |
| QUINTILES for prescribed quantity of methadone with lowest quintile as baseline | | | | | | | | |
| Quintile 2: 281- 700mg | **4 154** | 0.73 | 0.45-1.17 | 0.189 | 2.17 | 1.19-3.96 | 0.011 | **2 500** |
| Quintile 3: 701- 1200mg | **4 198** | 0.86 | 0.55-1.35 | 0.519 | 0.63 | 0.26-1.53 | 0.310 | **1 803** |
| Quintile 4: 1201- 1960mg | **4 734** | 1.10 | 0.72-1.67 | 0.660 | 1.20 | 0.60-2.38 | 0.611 | **2 210** |
| Quintile 5:  > 1960mg  (Eg, 1960mg=  14-days @ 140mg daily; or 28-days @ 70mg) | **4 646** | 1.43 | 0.95-2.14 | 0.084 | 2.76 | 1.50-5.09 | 0.001 | **1 625** |

*The* *GP-subset: quintiles for daily-dose versus quintiles for quantity; and options for their use in PH analysis of methadone-specific DRDs.*

**Table S5.** For the GP-subset, cross-tabulation of clients’ first CHI-identified prescription according to quintile of DAILY-DOSE and quintile of prescribed methadone (as defined by the GP-subset). Questionable combinations (238; 2%) are asterisked*.

| **GP-subset** | QUANTITY, Q1:  <=300 | QUANTITY, Q2:  301-840 | QUANTITY, Q3:  841-1400 | QUANTITY, Q4:  1401-2240 | QUANTITY, Q5:  > 2240 mg | **DAILY-DOSE TOTALS** |
| --- | --- | --- | --- | --- | --- | --- |
| DAILY-DOSE, Q1: <=30 | 1,324 | 865 | 87 | 29* | 45* | **2,350** |
| DAILY-DOSE,  Q2: 31-50 | 446 | 606 | 928 | 155 | 71 | **2,206** |
| DAILY-DOSE, Q3: 51-70 | 149 | 468 | 336 | 991 | 238 | **2,182** |
| DAILY-DOSE,  Q4: 71-90 | 104* | 191 | 391 | 584 | 574 | **1,844** |
| DAILY-DOSE, Q5: > 90 mg | 60* | 157 | 208 | **253** | **1,120** | **1,798** |
| **QUANTITY,**  **TOTALS** | **2,083** | **2,287** | **1,950** | **2,012** | **2,048** | **10,380** |

For the GP-subset of clients with known daily-dose at their first CHI-identified prescription, the highest quintile for daily-dose of prescribed methadone (> 90 mg) may be even more prognostic than for quantity prescribed, see right-most panel in **Table S6.**

**Table S6.** Proportional hazards analysis from first CHI-identified prescription to methadone-specific DRD for the subset of GP-clients for whom daily-dose of methadone is known at first CHI-identified prescription in July 2009 to June 2013: baseline covariates include quintile of prescribed quantity of methadone (as in **Table 2**), quintile of prescribed quantity of methadone (as defined by GP-subset) or quintile of daily-dose (as defined by GP-subset).

| Covariate | 10 380 GP-clients with daily-dose at first CHI-identified prescription who experienced 105 methadone-specific DRDs | | | | | | | | |
| --- | --- | --- | --- | --- | --- | --- | --- | --- | --- |
|  | Quintile for Quantity prescribed as defined in **Table 2** [1] | | | Quintile for Quantity prescribed as defined by GP-client subset [2] | | | **Quintile for DAILY-DOSE** **as** **defined by GP-client subset, see Table S5 [3]** | | |
|  | HR | 95% CI | p-value | HR | 95% CI | p-value | **HR** | **95% CI** | **p-value** |
| Female | 1.05 | 0.69-1.58 | 0.831 | 1.05 | 0.69-1.59 | 0.816 | **1.06** | **0.70-1.60** | **0.794** |
| Aged 35+ | 2.48 | 1.62-3.80 | <0.001 | 2.46 | 1.61-3.68 | <0.001 | **2.48** | **1.62-3.79** | **<0.001** |
| QUINTILE for prescribed methadone (quantity or DAILY-DOSE) with lowest quintile as baseline | | | | | | | | | |
| Quintile2 | 1.14 | 0.58-2.25 | 0.700 | 1.06 | 0.55-2.04 | 0.869 | **1.25** | **0.60-2.57** | **0.549** |
| Quintile3 | 0.54 | 0.24-1.24 | 0.147 | 0.67 | 0.32-1.40 | 0.284 | **1.77** | **0.91-3.47** | **0.094** |
| Quintile4 | 1.00 | 0.52-1.93 | 0.990 | 1.09 | 0.56-2.11 | 0.794 | **1.77** | **0.88-3.53** | **0.106** |
| Quintile5 | 1.37 | 0.75-2.49 | 0.304 | 1.56 | 0.84-2.87 | 0.158 | **2.47** | **1.28-4.76** | **0.007** |
